# Hepatitis C virus infection characteristics and treatment outcomes in Canadian immigrants

**DOI:** 10.1186/s12889-020-09464-0

**Published:** 2020-09-03

**Authors:** Curtis L. Cooper, Daniel Read, Marie-Louise Vachon, Brian Conway, Alexander Wong, Alnoor Ramji, Sergio Borgia, Ed Tam, Lisa Barrett, Dan Smyth, Jordan J. Feld, Sam Lee, on behalf of the CANUHC Cohort

**Affiliations:** 1 University of Ottawa, Roger Guindon Hall, 451 Smyth Rd #2044, Ottawa, ON K1H 8M5 Canada; 2 University of Ottawa, The Ottawa Hospital-General Campus, G12-501 Smyth Rd, Ottawa, ON K1H 8L6 Canada; 3 Laval University, Ferdinand Vandry Pavillon, 1050 Avenue de la Médecine, Quebec City, Quebec G1V 0A6 Canada; 4 Vancouver Infectious Diseases Centre, 1200 Burrard St, Vancouver, BC V6Z 2C7 Canada; 5 University of Saskatchewan, 107 Wiggins Rd, Saskatoon, SK S7N 5E5 Canada; 6 University of British Columbia, 317 - 2194 Health Sciences Mall, Vancouver, BC V6T 1Z3 Canada; 7 McMaster University, Michael DeGroote Centre for Learning and Discovery (MDCL) – 3104, 1280 Main Street West, Hamilton, ON L8S 4K1 Canada; 8 Liver Health Centre, 750 W Broadway, Vancouver, BC V5Z 1H2 Canada; 9 Dalhousie University, 5849 University Ave, Halifax, NS B3H 4R2 Canada; 10 Toronto General Hospital Research Institute, 200 Elizabeth St, Toronto, ON M5G 2C4 Canada; 11 Cumming School of Medicine, University of Calgary, 3330 Hospital Drive NW, Calgary, AB T2N 4N1 Canada

**Keywords:** Hepatitis C, Emigrants and immigrants, Antiviral drugs, Sustained Virological response

## Abstract

**Background:**

There are multiple obstacles encountered by immigrants attempting to engage hepatitis C virus (HCV) care and treatment. We evaluated the diversity and treatment outcomes of HCV-infected immigrants evaluated for Direct Acting Antiviral (DAA) therapy in Canada.

**Methods:**

The Canadian Network Undertaking against Hepatitis C (CANUHC) Cohort contains demographic information and DAA treatment information prospectively collected at 10 Canadian sites. Information on country of origin and race are collected. Characteristics and outcomes (sustained virological response; SVR) were compared by immigration status and race.

**Results:**

Between January 2016 and May 2018, 725 HCV-infected patients assessed for DAA therapy were enrolled in CANUHC (mean age: 52.66 ± 12.68 years); 65.66% male; 82.08% White, 5.28% Indigenous, 4.64% South East Asian, 4.64% East Indian, 3.36% Black). 18.48% were born outside of Canada. Mean age was similar [immigrants: 54.36 ± 13.95 years), Canadian-born: 52.27 ± 12.35 years); (*p* = 0.085)]. The overall baseline fibrosis score (in kPa measured by transient elastography) was similar among Canadian and foreign-born patients. Fibrosis score was not predicted by race or genotype. The proportion initiating DAA therapy was similar by immigrant status (56.72% vs 49.92%). SVR rates by intent-to-treat analysis were similar (immigrants-89.47%, Canadian-born-92.52%; *p* = 0.575).

**Conclusion:**

A diverse immigrant population is engaging care in Canada, initiating HCV antiviral therapy in an equitable fashion and achieving SVR proportions similar to Canada-born patients. Our Canadian experience may be of value in informing HCV elimination efforts in economically developed regions.

## Background

Hepatitis C Virus (HCV) afflicts around 71 million individuals worldwide and is responsible for an estimated 399,000 deaths per year [1]. Direct-acting antivirals (DAAs) consist of a short-duration, well tolerated oral drug regime which has proven to be a breakthrough in HCV treatment leading to sustained virologic response (SVR) rates of over 90% in patients with all viral genotypes [2]. Despite the ability of DAAs to reliably cure chronic infection, HCV remains wide-spread and underdiagnosed in Canada [3, 4]. Thus, HCV remains a serious threat to health and well-being with 32,500 Canadians projected to die from HCV-related liver complications between 2031 and 2035 [3].

Although 21.9% of Canada’s population was born elsewhere, immigrants and refugees to Canada represent 35% of HCV-infected individuals across the nation, with newcomers from endemic regions such as Sub-Saharan Africa, Asia and Eastern Europe presenting with a 1.5–1.7-fold higher HCV seroprevalence in comparison to native-born Canadians [4–6]. In addition, immigrants face many barriers to accessing healthcare including language barriers, unfavourable socioeconomic situations and cultural reservations; all of which may limit treatment uptake and/or completion [7, 8]. Failure to diagnose and treat would undoubtedly increase the risk for developing complications of HCV including hepatocellular carcinoma (HCC), which is already three-fold more common in Canadian immigrant populations [9, 10].

We assessed DAA treatment uptake and outcome between immigrants and Canada-born individuals utilizing patient demographic data collected at 10 treatment centres across Canada by the Canadian Network Undertaking against Hepatitis C (CANUHC) Cohort.

## Methods

### Population

The Canadian Network Undertaking against Hepatitis C (CANUHC) Cohort contains demographic and HCV DAA treatment information prospectively collected since January 2016 at 10 Canadian-based, publicly funding clinic sites at which patients are referred for HCV evaluation and antiviral treatment.

### Outcome measurements

Data is obtained by case report forms completed over the course of treatment including an intake, pre-treatment, post treatment non-SVR, post treatment SVR, and annual follow-up forms. SVR was defined as HCV RNA negativity 12 weeks or more following completion of DAA therapy. The intake form captures data including demographic information, medical and mental health comorbidities, risk factors for HCV infection, previous and current HCV treatment history, laboratory values (hemoglobin, platelets, INR, ALT, AST, bilirubin, albumin, sodium, creatinine, GFR), HCV genotype and viral load, fibrosis stage measured in kPa by transient elastography, and SVR12 status. HCV infection was defined as HCV RNA positivity more than 6 months after exposure. An immigrant was defined as any person residing in Canada who was born elsewhere. Information on immigration history, country of origin and self-reported race is collected. Remote versus recent year of immigration was defined before and after the calculated median year of arrival in Canada. Social determinants of health were based on employment history and self-reported alcohol use.

### Statistical analysis

Characteristics, DAA initiation, and antiviral treatment outcomes [SVR calculated by intent-to-treat (ITT) where all DAA dosed patients were included and on-treatment analysis where unknown SVR12 outcomes were excluded] were compared by immigration status and race using Student’s t test, Chi square and Fisher’s exact test where appropriate. New participant recruitment was censored in May 2018 but outcome data was gathered up to December 2019. Variables, which were selected based on mechanistic and biological grounds, included age, sex, race, genotype and fibrosis stage were input into the logistic model with an automated forward variable selection algorithm to find the best parsimonious model to predict SVR in the overall and immigrant population. Timing of arrival was considered in the SVR analysis limited to the immigrant population. To prevent overfitting of the model, the association of covariates with SVR had to have a significance level ≤ 0.01 to remain in the model. Fibrosis was evaluated as a function of age, biologic sex, race, immigrant status, genotype and diabetes status by linear regression analysis. Analysis was conducted using IBM SPSS Version 26.

### Ethical considerations

Participants attending CANUHC sites and providing written informed consent are enrolled. Overall research ethics board approval for this cohort study was obtained by the Health Research Ethics Board of Alberta (HREBA)-Community Health Committee (CHC) [HREBA.CHC-16-0038_REN3] where the primary database is housed. Each individual contributing site has also obtained local research ethics board approval.

## Results

Of 725 HCV-infected patients assessed for DAA treatment between January 2016 and May 2018, 18.48% (*n* = 134) were born outside of the country (Table 1). Among immigrants, 17.16% were born in South East Asia and the Indian Subcontinent and 6.72% originating from Sub-Saharan Africa. There was greater racial diversity in foreign-born patients. The mean age of participants was similar between groups with a mean of 54.36 ± 13.95 years) in foreign-born and 52.27 ± 12.35 years) in native-born patients. The median year of arrival for immigrants was 1996 (quartiles: 1981, 2004). Baseline laboratory measurements were similar between groups (Table 2).

**Table 1:** Baseline Characteristics and Direct Acting Antiviral Outcomes compared between Immigrant and Canadian Born Patients

	Immigrant to Canada (***n*** = 134) n (%)	Canadian Born (***n*** = 591) n (%)	***P*** Value
**Male Sex**	70 (52.24)	406 (68.70)	< 0.001
**Race**	*n* = 114	*n* = 511	< 0.001
White	53 (46.49)	460 (90.02)	
Black	10 (8.77)	11 (2.15)	
South East Asian	24 (21.05)	5 (0.98)	
East Indian	26 (22.81)	3 (0.59)	
Indigenous	1 (0.88)	32 (6.26)	
**Genotype**	*n* = 129	*N* = 571	< 0.001
1a	24 (18.60)	311 (54.47)	
1b	38 (29.46)	53 (9.28)	
1 other subtype	4 (2.33)	10 (1.75)	
2	13 (10.08)	59 (10.33)	
3	32 (24.81)	134 (23.47)	
4	13 (10.08)	2 (0.35)	
6	5 (3.88)	2 (0.35)	
**Fibrosis Stage**	*N* = 119	*N* = 523	
Stage 1	49 (41.18)	209 (39.96)	0.589
Stage 2	20 (16.81)	117 (22.37)	
Stage 3	18 (15.13)	71 (13.58)	
Stage 4 (Cirrhosis)	32 (26.89)	126 (24.09)	
**Currently Employed**	54 (40.30)	141 (23.86)	0.004
**Incarceration History**	11 (8.21)	154 (26.06)	< 0.001
**Received Blood Product**	19 (14.18)	64 (10.83)	0.293
**Alcohol (Past Use)**	52 (38.81)	218 (36.89)	0.693
**Alcohol (Current Use)**	40 (29.85)	177 (29.95)	> 0.999
**Recreational Drug Use (Ever)**	35 (26.12)	410 (69.37)	< 0.001
**Antiviral Treatment Naïve**	40/69 (57.97%)	244/345 (70.72%)	0.037
**Initiated a DAA Regimen**	76/134 (56.72%)	295/591 (49.92%)	0.155
**SVR (Intent-to-Treat)**	**68/76 (89.47%)**	**270/295 (91.52%)**	**0.575**

*DAA* Direct Acting Antiviral, *SVR* Sustained Virological Response

Some differences were seen in the proportion of concurrent co-morbidities between the two groups (Table 3). The immigrant population had a higher proportion of hepatocellular carcinoma (HCC) (2.99%) compared to native-born (0.68%, *p* = 0.042). Of note, this risk was not predicted by genotype 3 infection (data not shown). The immigrant population were less likely to be HIV co-infected (1.49%) compared to native-born (5.92%, *p* = 0.031) and to have psychiatric illness (11.19% vs. 23.86%, *p* = 0.001). Other concurrent co-morbidities were similar between populations including diabetes (10.45% vs. 6.43%, *p* = 0.135), chronic renal disease (0.75% vs. 0.51%, *p* = 0.559), and hemodialysis (0% vs. 0.34%, *p* > 0.999) in the immigrant versus the Canadian-born population, respectively.

**Table 2:** Baseline Laboratory Measures compared between Immigrant and Canadian Born Patients

	Immigrant to Canada Mean (SD)	Canadian Born Mean (SD)	***P*** Value
HCV RNA (IU/mL)	1.99 × 10^6^ (2.34 × 10^6^)	1.59 × 10^6^ (3.00 × 10^6^)	0.66
Hemogloblin (g/L)	137 (26)	140 (28)	0.190
Platelets (10^9^/L)	189 (74)	210 (79)	0.009
ALT (IU/L)	63 (62)	79 (110)	0.101
AST (IU/L)	53 (44)	64 (68)	0.084
Bilirubin (mmol/L)	11.7 (6.9)	11.4 (8.5)	0.785
Albumin (g/L)	40 (11)	42 (36)	0.588
Creatinine (mmol/L)	90 (102)	82 (61)	0.314
eGFR	99 (78)	102 (120)	0.808

*ALT* Alanine aminotransferase, *AST* Aspartate aminotransferase, *eGFR* Estimated glomerular filtration rate

**Table 3:** Concurrent Co-Morbidities compared between Immigrant and Canadian Born Patients

	Immigrant to Canada (***n*** = 134) n (%)	Canadian Born (***n*** = 591) n (%)	***P*** Value
Liver Transplant	0 (0)	1 (0.17)	> 0.999
Hepatocellular Carcinoma	4 (2.99)	4 (0.68)	0.042
Human Immunodeficiency Virus	2 (1.49)	35 (5.92)	0.031
Chronic Renal Disease	1 (0.75)	3 (0.51)	0.559
Diabetes	14 (10.45)	38 (6.43)	0.135
Hemodialysis	0 (0)	2 (0.34)	> 0.999
Psychiatric Illness	15 (11.19)	141 (23.86)	< 0.001

The prevalence of particular social determinants of health was distinct between the two populations (Table 1). Compared to Canadian-born patients, the immigrant population had a higher proportion of employment (40.30% vs. 23.86% *p* = 0.004), lower history of previous incarceration (8.21% vs. 26.06%, *p* = < 0.001) and present or past recreational drug use (26.12% vs. 69.37%, *p* = < 0.001). Other social factors were similar between both groups including past use of alcohol (38.81% vs. 36.89%, *p* = 0.69) and current use of alcohol (29.85 vs 29.95%, *p* > 0.999) in the immigrant and Canadian-born populations, respectively.

**Fig. 1:**
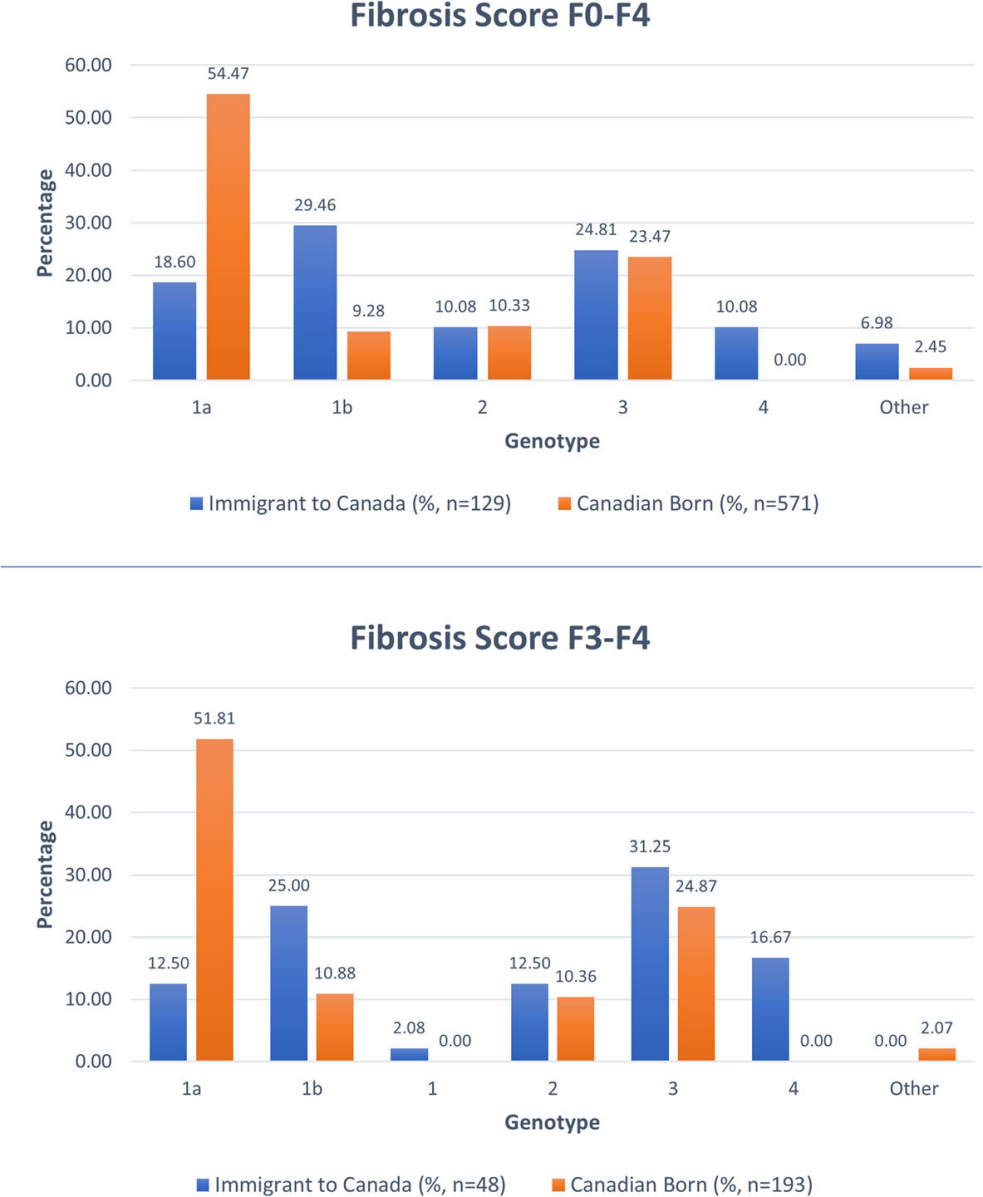
HCV genotype reflects country of origin in both the immigrant and Canadian born cohort (Fibrosis Stage 0–4) and is mirrored in the distribution of those patients of each cohort with advanced fibrosis (Fibrosis Stage 3–4)

We compared the genotype distributions between the immigrant and the native-born Canadian group (Table 1, Fig. 1). We also specifically assessed those with advanced fibrosis (defined as METAVIR Stage F3-F4). The genotype distributions differed between groups. However, we did not identify a difference in genotype proportions by immigrant-status between advanced fibrosis patients and patients at all stages of fibrosis. The immigrant group was more likely to be genotype 1b (from at least 25 different countries) and genotype 4 infected (6 of 13 from Egypt and 5 of 13 from Sub-Saharan Africa). The Canadian-born group was more likely to be genotype 1a infected. The genotype 2 and 3 proportions were similar by group and fibrosis stage categories. The mean HCV RNA levels were similar by group.

The median baseline fibrosis score determined by converted transient elastography score was fibrosis stage 2 (Table 1). Fibrosis stage was similarly distributed between the two groups (*p* = 0.589) with 41.18, 16.81, 15.13 and 26.89% of the immigrant group having a fibrosis score of F0–1, F2, F3 and F4. A fibrosis score distribution of 39.96, 22.37, 13.58 and 24.09% for F0–1, F2, F3 and F4 was observed in the Canadian-born group. Mean fibrosis score in kPa by transient elastography was similar by immigration status (11.0 kPa in immigrants vs 10.8 kPa in Canadian-born). By multivariable linear regression analysis, male sex, older age and diabetes (but not race or genotype) predicted higher fibrosis score by transient elastography (data not shown).

A total of 371 patients were documented to have initiated DAA regimens during the period of evaluation (Table 1). The proportions were numerically higher but statistically similar by immigrant status (56.72% in immigrants vs 49.92% in Canadian-born, *p* = 0.155). This did not differ between recent and remote year of arrival to Canada (data not shown). A greater proportion of the Canadian-born population were treatment naïve (70.72%) compared to the immigrant population (57.97%, *p* = 0.037). The most common DAA regimens prescribed during this period of evaluation included: sofosbuvir-velpatasvir (39.62%), ledipasvir-sofosbuvir (18.33%), elbasvir-grazoprevir (13.75%), ombitasvir-paritaprevir/ritonavir-dasabuvir (12.13%), and sofosbuvir-velpatasvir-voxilaprevir (2.70%). The prescribed regimens did not differ by immigrant status (*p* = 0.630). By intent-to-treat analysis, 89.47 and 91.52% of immigrants and Canadian-born DAA recipients, respectively, achieved SVR (*p* = 0.575). The results were similar by on-treatment analysis when one case was removed due to unknown week 12 post treatment HCV RNA result. Among the immigrant group, sex, race, recent versus remote year of immigration, and genotype did not predict SVR. However, the presence of cirrhosis did predict reduced SVR in the immigrant group (76.19% vs 93.47%, *p* = 0.043). By multivariable analysis of the entire cohort, lower SVR was predicted by the presence of cirrhosis but not race, genotype or sex (data not shown). Data related to lost-to-follow-up, DAA adherence, on treatment viral breakthrough, post treatment viral relapse, and re-infection were not available for analysis.

## Discussion

Immigrants to economically developed countries, including Canada, have a higher seroprevalence of HCV and are more likely to suffer from serious end-stage outcomes of chronic HCV infection compared to the Canadian-born population [9, 10]. The advent of DAAs provides great promise to this population as the first easily administered, well tolerated and highly curative treatment for HCV [2]. With this in mind, it is important to assess whether the numerous barriers to healthcare often faced by immigrants impede their ability to access, adhere to and achieve cure with DAA treatments [8].

We found that the proportion of immigrants initiating DAA treatment was similar to Canadian-born patients. In these patients, SVR rates did not differ based on immigrant status and were comparable to the high cure rates seen in clinical trials. Likelihood of DAA treatment initiation and SVR were similar whether recently or remotely immigrating to Canada. Previous studies in other healthcare domains have demonstrated that immigrant populations frequently face health care engagement obstacles including differences of language, culture, custom and socioeconomic status as compared to the population of the host nation [7, 8, 11]. However, our DAA analysis is consistent with previous HCV-specific analyses identifying no distinct difference between SVR rates in Canadian-born and immigrant populations for both interferon-based and DAA treatments [12, 13]. Universal access to the publically funded Canadian health care system and full reimbursement for DAA treatment facilitated engagement and retention in HCV care, treatment and cure. Our analysis, further described below, suggest that HCV represents an unusual situation in which the non-immigrant population is characterized by a very heavy burden of psycho-socioeconomic challenges. This should be considered when interpreting the relatively good outcomes of those born outside of Canada.

We found that immigrants were less likely than Canadian-born patients to report substance abuse, previous incarceration, mental health illness and HIV co-infection. The finding of higher drug use amongst Canadian-born patients is consistent with literature suggesting that individuals from high income countries are most likely to contract HCV by injection drug use whereas individuals born in developing countries more commonly contract HCV through iatrogenic transmission with unsterile medical equipment [1, 10, 14–16]. Our observation of higher previous incarceration rates in the Canadian-borne versus immigrant population is not surprising since HCV is reported to have a seroprevalence amongst Canadian federal inmates of as high as 24.0% (versus 0.96% for Canada as a whole) [4]. Furthermore, a history of incarceration often precludes successful immigration to economically developed countries. The finding of higher HIV co-infection in the host-population is consistent with previous studies and is likely in itself related to the higher rates of drug use and incarceration recorded in this study which have been identified as risk factors for HIV-HCV co-infection [10, 12, 13, 17].

The impact of unemployment on HCV treatment uptake and cure is conflicting. Some evaluations have suggested that unemployment and low socioeconomic status can present a barrier to treatment [7, 8]. One study by Ahmed et al. suggested that new immigrants often need to take on several low-paying jobs concurrently in order to provide for their families, potentially causing interruption or discontinuation of treatment from missed appointments due to work [8]. However, Giordano et al. identified no difference in SVR with interferon-based HCV treatment between immigrants and Canadian-born patients despite a lower employment rate amongst the former [13]. In our cohort, immigrants had a higher rate of employment than the Canadian-born patient cohort. It would seem that rates of unemployment alone are not necessarily indicative of a barrier, or a lack thereof, to treatment and cure. We were limited by the fact that we did not acquire complete data about the type or quality of employment or medical benefits, which may have provided a more accurate picture of socioeconomic stability in both groups. This presents a possible avenue for future inquiry.

Like many economically developed nations, Canada is a diverse country with over one-fifth of Canadians identifying as a visible minority [5]. Discrimination in the healthcare system would have a far-reaching impact. Our finding that SVR with DAA treatment was not diminished in immigrants or in any race is reassuring given our aim for equal access to treatment in Canada. We do recognize that our analysis was limited in that it focused only on those who successfully engaged in HCV care and initiated treatment. With this in mind, it is plausible that both our Canadian-born and immigrant populations may have been influenced by selection bias.

We observed that 17% of our immigrant patient group originated from South East Asia and the Indian Subcontinent and 7% originated from sub-Saharan Africa. Previous analyses have reported that migrants from South Asia and sub-Saharan Africa displayed HCV seroprevalence greater than 3% and identified those from Asia, sub-Saharan Africa and Eastern Europe as high prevalence groups for HCV infection [18]. The high frequency of HCV infection amongst this demographic observed in our study is further evidence in support of routine screening of immigrants [6, 10, 12, 19]. Increased screening would in part address the above discussed selection bias pertaining to HCV treatment engagement.

There are strengths and limitations to this investigation that should be considered in addition to those mentioned above. Our cohort was derived from multiple clinics from across Canada including large HCV programs in Canada’s five most populous and diverse urban cities. We this in mind, we believe that our results are representative of the greater population of people living with HCV in Canada. We did not have full data on year of HCV infection or diagnosis. Additional detail related to initial engagement in HCV treatment programs, adherence, language barrier, on- and post-treatment loss to follow up and viral failure/relapse would have been illuminating to help fully understand why a fairly large proportion of both immigrant and Canadian-born individuals did not initiate DAA therapy during the period of evaluation and to understand why some patients did not achieve SVR. Unfortunately, this level of detailed information was not available in this dataset.

## Conclusions

We identified similarities and key differences between Canadian-born and foreign-born HCV patients in our cohort. The two populations were distinct in terms of race, co-morbidities and viral characteristics. Despite these discrepancies, we found that these two HCV treatment program-engaged populations experienced similar high HCV cure rates. With this in mind, we believe that these Canadian-specific findings may be of value in informing HCV elimination efforts in other diverse, economically developed nations.

## Data Availability

The datasets generated and/or analysed during the current study are not publicly available due to privacy concerns but are available from the corresponding author on reasonable request.

## Abbreviations

HCV: Hepatitis C
DAA: Direct Acting Antiviral
CANUHC: Canadian Network Undertaking against Hepatitis C
SVR: Sustained Virologic Response
kPa: Kilopascal
HIV: Human Immunodeficiency Virus
HCC: Hepatocellular Carcinoma
INR: International Normalized Ratio
ALT: Alanine Aminotransferase
AST: Aspartate Aminotransferase
GFR: Glomerular Filtration Rate
ITT: Intent-to-Treat
SD: Standard Deviation
RNA: Ribonucleic Acid
